# Targeting G-quadruplex motifs interferes with differentiation of adipose-derived mesenchymal stem cells

**DOI:** 10.1186/s13287-023-03320-9

**Published:** 2023-04-19

**Authors:** Maria Rosaria Ambrosio, Teresa Migliaccio, Fabiana Napolitano, Sarah Di Somma, Giovanni Maneli, Jussara Amato, Bruno Pagano, Antonio Randazzo, Giuseppe Portella, Pietro Formisano, Anna Maria Malfitano

**Affiliations:** 1 Department of Translational Medical Sciences, University “Federico II”, 80131 Naples, Italy; 2 URT Genomics of Diabetes, Institute of Experimental Endocrinology and Oncology, National Research Council, 80131 Naples, Italy; 3 Department of Pharmacy, University of Naples Federico II, 80131 Naples, Italy

**Keywords:** G-quadruplex, Mesenchymal stem cells, Differentiation, Adipocytes

## Abstract

**Background:**

G-quadruplex (G4) motifs are nucleic acid secondary structures observed in mammalian genomes and transcriptomes able to regulate various cellular processes. Several small molecules have been developed so far to modulate G4 stability, frequently associated with anticancer activity. However, how G4 structures are regulated over homeostatic conditions is mostly unexplored. Here, we used human adipose-derived mesenchymal stem cells (ASCs) to address the role of G4 motifs during adipogenic differentiation.

**Methods:**

Adipocyte differentiation of ASCs was investigated in the presence or absence of a well-known G4 ligand, Braco-19. Cell viability was determined by sulforhodamine B assay. Cell dimension and granularity, DNA G4 motifs and cell cycle were detected by flow cytometry. Lipid droplet accumulation was assessed by Oil Red O staining. Cell senescence was evaluated by β-galactosidase staining. Gene expression was measured by qPCR. Protein release in the extracellular medium was quantified by ELISA.

**Results:**

Braco-19 used at non-cytotoxic concentrations induced morphological changes in mature adipocytes partially restoring an undifferentiated-like status. Braco-19 reduced lipid vacuolization and *PPARG*, *AP2*, *LEP* and *TNFA* mRNA levels in terminally differentiated cells. No effect was observed in cell senescence, fibrotic markers, IL-6 and IL-8 production, while the secretion of VEGF was dose-dependently reduced. Interestingly, G4 structures were increased in differentiated adipocytes compared to their precursors. Braco-19 treatment reduced G4 content in mature adipocytes.

**Conclusions:**

Our data highlight a new role of G4 motifs as genomic structural elements related to human ASC differentiation into mature adipocytes, with potential implications in physio-pathological processes.

## Background

G-quadruplex (G4) motifs are dynamic secondary structures of nucleic acids characterized by the formation of G-tetrads, planar cyclic arrays of four guanine bases linked by Hoogsteen hydrogen bonds [1]. G4 structures are evolutionary conserved and particularly enriched in telomeres, recombination sites and promoter regions of proto-oncogenes, such as *c-MYC, BCL-2, KRAS* and *c-KIT* [2].

Several studies addressed G4 motif stabilization by using selective small molecules to induce anticancer effects mainly associated with DNA damage at telomeres, cancer cell senescence, apoptosis, immunogenic cancer cell death [3, 4]. G4 structure stabilization blocks telomerase activity [5] and its access to the G-rich single strand, thus preventing telomere extension and cancer cell immortality. Targeting G4 motifs with specific ligands represents the focus of several clinical investigations in cancer. To this aim, several classes of G4 binders have been developed so far [6].

However, the presence of G4 structures in genomes and transcriptomes of all cell types and their roles in a series of biological processes, like transcription, replication, recombination and maintenance of chromosome stability, suggests their involvement in other cell functions like physiologic development and differentiation.

In line with this, a G4-forming sequence was detected in the promoter of Uncoupling Protein-1 (*UCP1*), a mitochondrial thermogenic gene, induced during progenitor cell differentiation toward brown or “brite” cells [7]. The presence of the G4 motif has been suggested to play a role in the regulation of *UCP1* expression, since 5,10,15,20-tetra(*N*-methyl-4-pyridyl) porphyrin (TMPyP4), likely destabilizing G4 in the promoter region, enhanced its transcription [7].

DNA G4 motifs were also detected in the promoter region of *c-kit*, a spermatogonia differentiation-related gene. It was demonstrated that in mouse testis, a G4 DNA resolvase, the RNA helicase associated with AU-rich element (RHAU), activated spermatogonia differentiation directly by binding to the G4 motifs in *c-kit* promoter [8].

Overall, these studies suggest a role of G4 motifs in cell differentiation. A very recent study demonstrated the loss of G4s during the differentiation of pluripotent human embryonic stem cells (hESC) into multipotent stem cells with differing lineage potential, cranial neural crest cells and neural stem cells [9].

Therefore, defining the regulation of G4 motif formation or loss during differentiation process is relevant and deserves further investigation. Studies dealing with how G4 motifs and their ligands might regulate normal cell functions and differentiation under homeostatic conditions are completely lacking in mesenchymal stem cells (MSCs). In this study, we used mammary adipose tissue-derived MSCs (ASCs) to address the role and function of G4 motifs in the adipogenic process. We investigated the presence of G4 motifs and their modulation by a well-known G4 binder, Braco-19 [10, 11], during adipocyte differentiation of ASCs.

## Methods

### Cell cultures

Adipose-derived mesenchymal stem cells (ASCs) were previously isolated from human adipose tissue biopsies [12, 13]. Cells were cultured in DMEM-F12 (1:1) supplemented with 10% FBS, 2 mM glutamine, 100 unit/ml penicillin and 100 unit/ml streptomycin. Cultures were maintained in a humidified atmosphere of 95% air and 5% CO_2_ at 37 °C. Media, sera and antibiotics were from Lonza (Basel, Switzerland). For cell viability and Oil Red O assays cells were cultured in 96 multiwell plates. For RNA isolation, flow cytometry assays, SA-b-gal staining and ELISA cells were cultured in 24 multiwell plates. For all the experiments, cell density was 3 × 10^4^ cells/cm^2^. Cells were routinely screened for mycoplasma contamination.

### Adipocyte differentiation and Oil Red O staining

ASCs were inducted to differentiate into adipocytes (ADIPO) by the alternation (every three days, two times) of an Adipocyte differentiation Induction Mix (850 nM Insulin, 10 μM Dexamethasone, 0.5 mM 3-IsoButyl-1-MethylXanthine, 33 μM Biotin, 17 μM Pantothenate and 1 μM Rosiglitazone) and an Adipocyte differentiation Maintaining Mix (850 nM Insulin and 1 μM Rosiglitazone). Then, the cells were stimulated with 1 μM Rosiglitazone. All the process was carried out in complete culture medium in the presence and absence of Braco-19 (0.15 to 10 µM). Adipocyte differentiation was reached in 13 days and lipid accumulation determined by Oil red O staining. Stained oil droplets were dissolved with isopropanol and evaluated as optical density at 490 nm by Glomax Discover Microplate Reader (Promega, Madison, WI, USA). Images of stained monolayers were taken by the Olympus DP20 microscope digital camera system (Olympus Corporation, Tokyo, Japan). All chemicals were from Sigma-Aldrich (MO, USA).

### Cell viability assay

Cells were fixed with 50% trichloroacetic acid for at least 2 h at 4 °C, washed with distilled and de-ionized water, air-dried and stained 30 min with 0.4% sulforhodamine B (SRB) in 1% acetic acid. Unbound dye was removed, and 10 mM Tris solution (pH 7.5) was added to dissolve the protein-bound dye [14, 15]. Cell survival was assessed by optical density determination at 490 nm by Glomax Discover Microplate Reader (Promega, Madison, WI, USA). All chemicals were from Sigma-Aldrich (MO, USA). Images before fixing the cells were taken by the Olympus DP20 microscope digital camera system (Olympus Corporation, Tokyo, Japan).

### RNA isolation, RT-PCR and qPCR

Total RNA was isolated using TRIzol solution according to the manufacturer’s instructions. RNA samples were quantified by measuring the absorbance at 260 and 280 nm (NanoDrop spectrophotometer, Life Technologies). RNA samples were reverse transcribed using SuperScript III Reverse Transcriptase with oligo dT primers according to the manufacturer’s instructions. To check the amplifiable template RNA/cDNA, RT-PCR amplification of housekeeping genes was performed. Amplification reactions were set up using AmpliTaq Gold and specific primer pairs, designed by Oligo 4.0 (Table 1). qPCR was performed by iTaq Universal SYBR Green Supermix, according to the manufacturer’s instructions for the CFX Connect Real-Time system (Biorad). Relative quantification of gene expression was measured by using 2 − ΔΔCt method. Expression levels were normalized for the reference sample using peptidylprolyl isomerase A (PPIA) as housekeeping gene. TRIzol solution for RNA isolation, SuperScript III Reverse Transcriptase with oligo dT primers for RNA reverse transcription and AmpliTaq Gold for RT-PCR were from Life Technologies (Carlsbad, CA, USA). iTaq Universal SYBR Green Supermix for quantitative real-time PCR (qPCR) was from Biorad (Hercules, CA, USA). The primer sequences used for the gene expression analysis are listed in Table 1.

**Table 1:** Primers

GENE	Forward	Reverse	*T*_m_ (°C)	Size (bp)
PPARG	5′-GAGAAGGAGAAGCTGTTGGC-3′	5′-ATGGCCACCTCTTTGCTCT-3′	60	272
LEPTIN	5′-GTGCCCATCCAAAAAGTCCA-3′	5′-GGAAGGCATACTGGTGAGGA-3′	60	210
AP2	5′-TGTGTGATGCTTTTGTAGGTAC-3′	CTTCGTCAAATTCCTGGCCC-3′	60	215
LMNB1	5′-GCCCAGATCAAGCTTCGAGA-3′	5′-GCTTCCAACTGGGCAATCTG-3′	60	134
ACTA2	5′-AGAACATGGCATCATCACCA-3′	5′-GAGTCATTTTCTCCCGGTTG-3′	60	149
FSP1	5′-CCACAAGTACTCGGGCAAAG-3′	5′-TGGGCTGCTTATCTGGGAAG-3′	60	248
TNFα	5′-GGCTTGTCACTCGGGGTT-3′	5′-GGGACCTCTCTCTAATCAGCC-3′	60	70
IL-6	5′-CAATGAGGAGACTTGCCTGGT-3′	5′-AGCTGCGCAGAATGAGATGA-3′	60	275
IL-8	5′-TGAGAGTGATTGAGAGTGGA-3′	5′-TCAAAAACTTCTCCACAACCC-3′	60	275
VEGF	5′-TCACAGGTACAGGGATGAGGACAC-3′	5′-TCCTGGGCAACTCAGA-3′	60	184
PPIA	5′-TACGGGTCCTGGCATCTTGT-3′	5′-GGTGATCTTCTTGCTGGTCT-3′	60	196

### Forward scatter (FSC) and side scatter (SSC) analyses, cell cycle and G4 structure staining

Cells were detached with trypsin and washed twice with PBS. Cells were fixed in 70% (v/v) ethanol and stored at − 20 °C overnight. Cell pellet was washed with PBS/Tween buffer (PBT) (0.5% w/v BSA and 0.1% v/v Tween 20 in PBS) and re-suspended in PBT with anti-G4 BG4 antibody (Millipore, Burlington, MA, USA, MABE 917, 1:100). Cells were incubated 1 h at room temperature, washed with PBT and re-suspended in PBT containing Alexa Fluor 488 (Invitrogen, USA, #A11001, 1:100) at darkness. After 30 min, cells were washed with PBT containing RNaseA (Roche) (0.4 U) and re-suspended in PI (Sigma) (0.015 mol/L) for 20 min at room temperature [15].

The flow cytometry acquisition settings were as follows: First, a FCS area/SSC area (FSC-A/SSC-A) dot plot was drawn to verify that the population of interest was appropriately displayed or required to adjust voltages. Then, a FSC height (FSC-H) vs FCS area (FSC-H/FSC-A) dot plot was used to define and gate on single cells and remove artifacts, doublets and aggregates. FSC and SSC were analyzed in unstained cells as mean fluorescence intensity (MFI) signal. For cell cycle analysis, the singlet region selected in FSC-H/FSC-A dot plot to exclude doublet was used to apply a PI histogram plot (PI vs cell counts) [16, 17]. The PI emission was measured with a suitable bandpass filter 610/20. Markers identifying each cell cycle phases (subG0/G1, G1, S, G2/M) were settled for each experimental condition. G4 motif quantification was performed on the singlet region by drawing a dot plot FITC vs PI. On PI stained cells, FITC + cells were gated as representing G4 motif content. Flow cytometry acquisitions were performed with a BD LSRFortessa (BD Biosciences, San Jose, CA, USA) and analyses performed using Flowlogic Software (MACS, Miltenyi Biotec).

### SA-b-gal staining

Cell senescence was assessed by SA-b-gal Staining kit (Cell BioLabs, San Diego, CA, USA). Cells were washed twice with PBS (1x) and left at room temperature for 5 min with 1 × Fixing Solution. Cells were washed three times with PBS (1x) and then completely covered with freshly prepared Cell Staining Working Solution in the dark for 4 h at 37 °C. After the incubation, the solution was removed, the cells were washed twice with PBS (1x) and blue-stained senescence cells were observed by the Olympus DP20 microscope digital camera system (Olympus Corporation, Tokyo, Japan).

### ELISA assays

TNF-α, IL-6, IL-8 and VEGF secretion levels were measured in conditioned media collected from ADIPO. ELISA assays were assessed according to manufacturer’s instructions of Human TNF-α, Human IL-6, Human IL-8 (ELISA, Thermo Fisher) and Human VEGF (DuoSet ELISA, RD system) kits.

### Statistical analysis

Statistical analyses were carried out using GraphPad Prism 7 (GraphPad Software). Differences among groups were evaluated by ANOVA corrected for multiple comparison tests or by parametric unpaired two-tailed T test or the nonparametric Mann–Whitney test for pairwise comparisons (as indicated in the legends of the figures). *P* values < 0.05 were considered statistically significant.

## Results

### Effect of Braco-19 on ASC viability and cell cycle

**Fig. 1:**
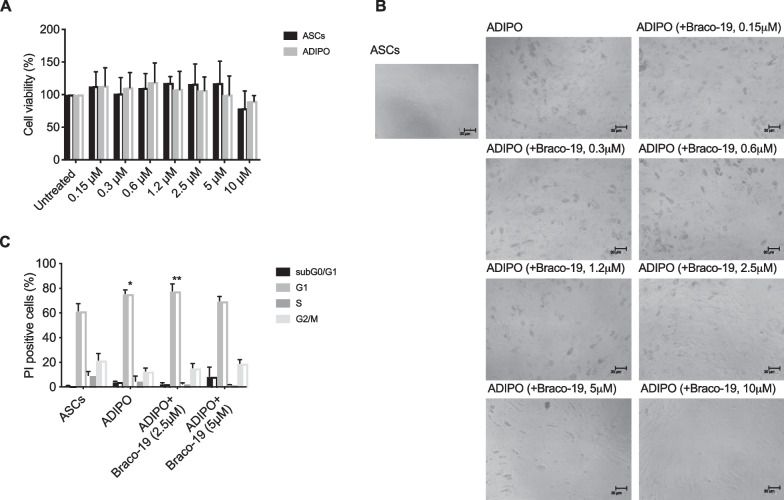
Effect of Braco-19 on ASCs: survival, morphology and cell cycle. Undifferentiated ASCs and differentiating ASCs (ADIPO; gray bars) were treated with Braco-19 (0.15, 0.3, 0.6, 1.2, 2.5, 5, 10 µM) to determine cell viability by SRB assay. Results are reported as percentage of viable cells compared to untreated ASCs/ADIPO, considered as maximum viability (100%). Data represent the mean ± SD of at least three independent experiments performed in triplicate (**A**). Representative microscopic images (× 10 magnification, scale bar 30 µm) were taken at the end of the cultures (**B**). ASCs, ADIPO and ADIPO treated with Braco-19 (2.5 or 5 µM) were stained with PI to evaluate cell cycle. Bars in the graph represent the percentage of cells in subG0/G1, G1, S and G2/M phases (**C**). Data were analyzed using two-way ANOVA with Turkey’s multiple comparisons test (*p < 0.05; **p < 0.005)

To address potential cytotoxic activity, Braco-19 (from 0.16 to 10 µM) was added to both undifferentiated and differentiating ASCs (ADIPO). As shown in Fig. 1A, Braco-19 did not significantly affect ASC viability (Fig. 1A). However, morphological changes resembling undifferentiated ASCs were observed in ADIPO obtained by differentiating the cells in the presence of Braco-19, in a dose-dependent manner (Fig. 1B).

As expected, an increase in the G1 phase was detected in ADIPO, likely due to a blunted proliferation rate of differentiated cells; interestingly, Braco-19 at 5 µM, but not at lower doses, prevented G1 accumulation, partially restoring the undifferentiated profile (Fig. 1C).

### Effect of Braco-19 on adipocyte differentiation of ASCs

**Fig. 2:**
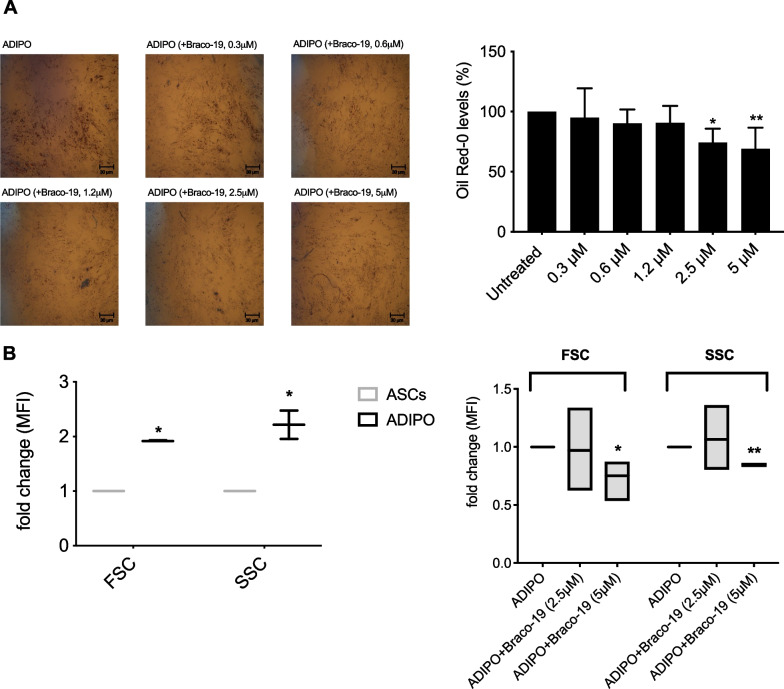
Effect of Braco-19 on adipocyte differentiation of ASCs. ADIPO were obtained by differentiating ASCs in the presence and absence of Braco-19 (0.3, 0.6, 1.2, 2.5, 5 µM). Lipid accumulation was determined by Oil Red O staining. Representative microscopic images (× 10 magnification) are reported on the left. Bar graph (on the right) shows Oil Red O quantification as percentage of Oil Red O levels in the presence of Braco-19 compared to untreated cells, considered as maximum differentiation (100%). Data (mean of at least three independent experiments) were analyzed using one-way ANOVA for multiple comparison with Dunnett method (*p < 0.05; **p < 0.01) (**A**). FSC and SSC were analyzed within the flow cytometry acquisitions of the cell cycle for ASCs, ADIPO and ADIPO treated with Braco-19 (2.5 or 5 µM). Data are reported as boxplots (mean of three independent experiments) showing the mean fluorescence intensity (MFI) as fold-change of ADIPO compared to ASCs (left graph), and ADIPO treated with Braco-19 compared to untreated ADIPO (right graph) (**B**). Statistical analysis was carried out by one unpaired T test per row correct for multiple comparisons using the Holm–Sidak method (left panel, *p < 0.0001; right panel,*p < 0.05;**p < 0.00001)

To further investigate the impact of Braco-19 on adipocyte differentiation, we quantified lipid droplet accumulation at the end of differentiation process carried out in the presence of Braco-19 at increasing doses (from 0.3 to 5 µM). As shown in Fig. 2A, lipid accumulation was significantly reduced in the presence of 2.5 and 5 µM Braco-19 compared to untreated ADIPO, and thus, these concentrations were used in the following assays. Flow cytometry analysis showed an increase in cell dimension (FSC) and granularity (SSC) in ADIPO compared to ASCs (Fig. 2B, left graph). The treatment of ADIPO with Braco-19 (5 µM) significantly reduced both FSC and SSC (Fig. 2B, right panel).

**Fig. 3:**
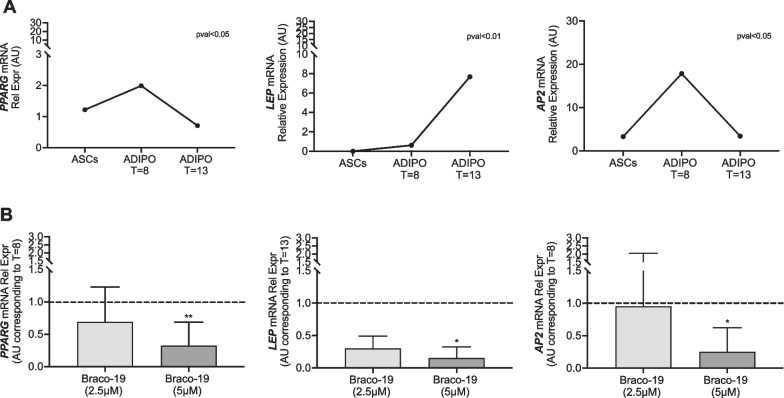
Effect of Braco-19 on adipogenic markers during adipocyte differentiation of ASCs. ASCs were differentiated toward the adipogenic lineage in the presence and absence of Braco-19 (2.5 or 5 µM). At different time point (day 8—T8—or day 13—T13) from the induction of adipocyte differentiation, mRNA levels of adipogenic genes (*PPARG, LEP, AP2*) were determined by qPCR. Data were normalized on *PPIA* gene as internal standard. Results are represented as mean ± SD of four independent experiments. **A** shows mRNA levels of *PPARG, LEP* and *AP2* in differentiating and terminally differentiated ADIPO (T8 and T13) relative to those in undifferentiated ASCs, in the absence of Braco-19. Panel **B** shows *PPARG* and *AP2* mRNA levels in ADIPO (T8), while *LEP* in ADIPO (T13) treated with Braco-19 relative to those in untreated ADIPO (T8 or T13, respectively) (dotted line). Data were analyzed using the nonparametric Mann–Whitney test for pairwise comparisons (*p < 0.05; **p < 0.01)

Then, we measured mRNA levels of adipogenic genes *PPARG, AP2* and *LEP* at different time points of adipocyte differentiation (day 8 and day 13, corresponding to differentiating and terminally differentiated ADIPO, respectively) both in the absence and in the presence of Braco-19 (2.5 and 5 µM). At first, we observed a significant increase in *PPARG* and *AP2* mRNA levels at day 8 while of *LEP* at day 13 of the adipogenic process (Fig. 3A). Interestingly, we found that 5 µM Braco-19 significantly reduced *PPARG* and *AP2* mRNA levels of about 7 and 7.5 fold, respectively, compared to untreated cells at day 8; similarly, a significant reduction of about tenfold was observed for *LEP* mRNA levels compared to untreated cells at day 13 (Fig. 3B). These results represent the first evidence that a G4 binder interferes with ASC differentiation toward the adipocyte lineage.

### Effects of Braco-19 on fibro-senescence features in adipocytes

**Fig. 4:**
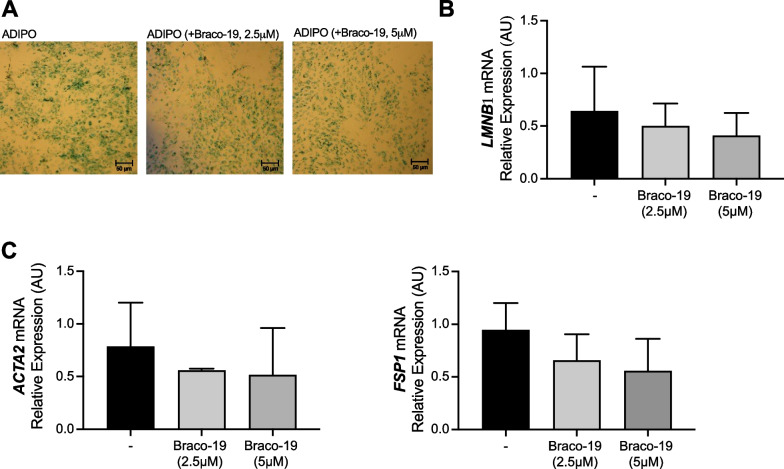
Effect of Braco-19 on fibro-senescence features in adipocyte differentiated from ASCs. ASCs were differentiated toward the adipogenic lineage (ADIPO) in the presence and absence of Braco-19 (2.5 or 5 µM). At the end of the process, blue Sa-b-Gal-positive senescent cells were detected by microscopy (× 10 magnification). Images are representative of at least three independent experiments (**A**). mRNA levels of senescence and fibrosis markers *LMNB1* (**B**), *ACTA2* and *FSP1* (**C**) were determined by qPCR. mRNA levels were normalized on *PPIA* gene as internal standard. Results are represented as mean ± SD of three independent experiments and show *LMNB1, ACTA2* and *FSP1* expression levels in cells treated with Braco-19 relative to those in untreated cells

We evaluated the effect of Braco-19 on senescence and fibrosis of ADIPO. As hallmarks of senescence, we measured β-gal activity and *LMNB1* mRNA levels. We observed that terminally differentiated ADIPO showed a markedly senescent phenotype, as indicated by the massive presence of blue β-gal-stained cells; Braco-19 did not induce detectable changes (Fig. 4A). Accordingly, although inducing a slight reduction, Braco-19 did not significantly affect mRNA levels of *LMNB1* in ADIPO (Fig. 4B). We also found that Braco-19 did not significantly modify mRNA levels of pro-fibrotic markers *ACTA2* and *FSP1* in ADIPO (Fig. 4C). Overall, such results indicate that Braco-19 has only a slight effect on fibro-senescence features in ADIPO.

### Effect of Braco-19 on pro-inflammatory molecules during adipogenesis

**Fig. 5:**
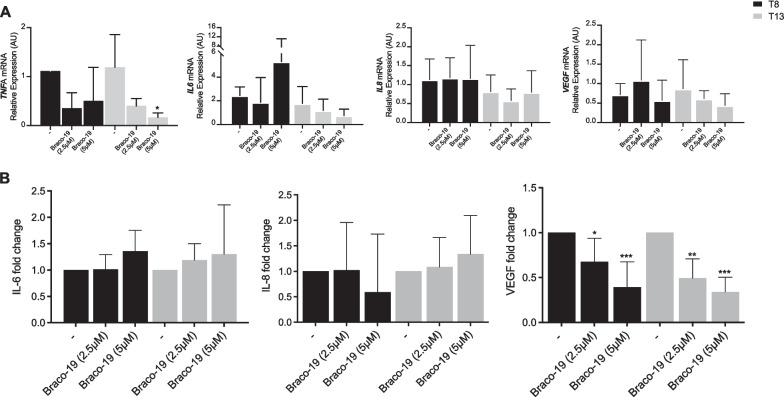
Effect of Braco-19 on inflammation molecules during adipocyte differentiation of ASCs. ASCs were differentiated toward the adipogenic lineage (ADIPO) in the presence and absence of Braco-19 (2.5 or 5 µM). Cells and conditioned media were collected at different time points (T8 or T13, black or gray bars, respectively) from the induction of adipocyte differentiation. mRNA levels of *TNFA, IL-6, IL-8* and *VEGF* were determined by qPCR and normalized on *PPIA* gene as internal standard. Results are represented as mean ± SD of three independent experiments (*p < 0.05) (**A**). Cytokine levels of IL-6, IL-8 and VEGF were measured by ELISA in conditioned media. Results are represented as mean ± SD of five independent experiments (**B**). The statistical analysis was performed by two-way ANOVA for multiple comparison with Dunnett method (*p < 0.05; **p < 0.001; ***p < 0.0001)

Next, we evaluated the effects of Braco-19 on pro-inflammatory molecules known or expected to be produced by ASCs and ADIPO [18]. Specifically, we chose molecules characterizing a senescence-associated secretory phenotype (SASP) to evaluate if their modulation could account for the slight reduction of *LMNB1* observed (Fig. 4B). We observed that Braco-19 (5 µM) significantly reduced *TNFA*, thus suggesting anti-inflammatory properties of this molecule in ADIPO. However, *IL-6* and *IL-8* were unmodified, while *VEGF* mRNA levels were slightly, although not significantly, reduced by Braco-19 (Fig. 5A). We further investigated the extracellular release of TNF-α, IL-6, IL-8 and VEGF. Secretion levels of TNF-α were undetectable, and IL-6 and IL-8 were not modulated by Braco-19, while VEGF was dose-dependently decreased (Fig. 5B). These results are in agreement with the slight effect on senescence above described, which only significantly reflects VEGF reduction, without deeply affecting the production of inflammatory cytokines. The large error bars observed in Fig. 5 are likely due the use of primary cells and to the different donors included in the study. Intriguingly, both the promoters of *TNFA* and *VEGF* are known to form G4 structures [19, 20]. Thus, we investigated the presence of G4 motifs during adipogenesis.

### Braco-19 reduces G4 motif formation in terminally differentiated ASCs

**Fig. 6:**
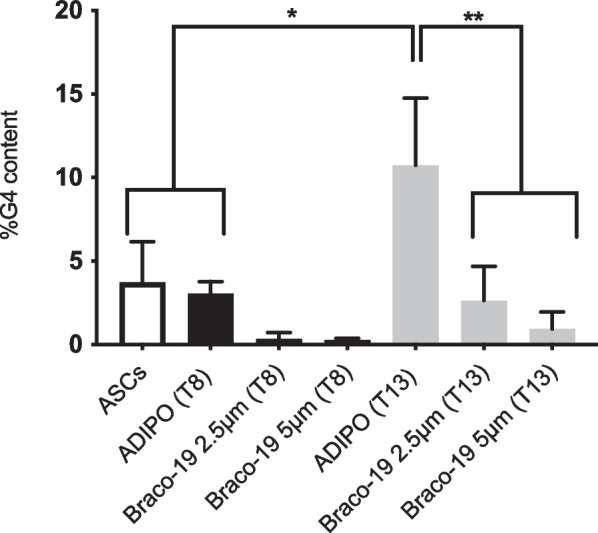
Effect of Braco-19 on G4 motif abundance in differentiated adipocytes. ASCs were differentiated toward the adipogenic lineage (ADIPO) in the presence and absence of Braco-19 (2.5 or 5 µM). At different time point (T8 or T13) from the induction of adipocyte differentiation, G4 motif formation was evaluated by flow cytometry in ASCs (white bar) and ADIPO (T8 or T13, gray or black bars, respectively). Bar graph reports the percent of G4 motif content (mean ± SD of three independent experiments). Data were analyzed by one-way ANOVA with Turkey’s multiple comparisons test (*p < 0.05; **p < 0.005)

We evaluated the presence of G4 structures and the effect of Braco-19 (2.5 and 5.0 µM) on undifferentiated (ASCs), differentiating (ADIPO at day 8) and terminally differentiated (ADIPO at day 13) cells. As shown in Fig. 6, terminally differentiated cells displayed significantly higher G4 motif contents compared to both ASCs and ADIPO at day 8. Surprisingly, Braco-19 reduced G4 motif amount in ADIPO at day 8 and day 13; however, the effect was statistically significant only at day 13 (Fig. 6). To the best of our knowledge, this result represents the first evidence that G4 motifs are related to adipogenic differentiation and that Braco-19 can destabilize G4 structures in adipose cells.

## Discussion

Beyond the reported potential of G4 motifs as anticancer targets [15, 21], studies dealing with how these secondary structures and their ligands might regulate normal cell functions under homeostatic conditions are poorly investigated. G4 structures detected throughout mammalian genome regulate several processes associated with gene expression and genome duplication. Few studies suggest a potential role of G4 motifs in cell differentiation [9]; however, how G4 motif regulation occurs during cell commitment toward specific lineages is completely unexplored.

Our study approaches the G4 motif landscape in human ASC differentiation toward mature adipocytes. Human ASCs represent attractive candidates for clinical use considering their hypo-immunogenic profile, their anti-inflammatory and pro-regenerative capacities [22–25]. Clinical trials based on the use of MSCs cover a wide variety of diseases ranging from acute and chronic inflammatory tissue deterioration in both humans and animals [26]; thus, a better understanding of mechanisms regulating ASC survival and differentiation is required to improve their biological function. Moreover, both the easy isolation and the extensive proliferation/differentiation potential make ASCs a suitable primary cell model to investigate in our study.

Among small molecules developed to bind and stabilize G4 motifs, Braco-19, a tri-substituted acridine compound, has been widely investigated in cancer cells. Braco-19 activity was ascribed to telomere shortening and induction of cell death mechanisms in cancer cells [27]. We have observed that Braco-19 activity in breast cancer cells is mediated by mechanisms ranging from apoptosis to immunogenic cell death, depending on the invasiveness of tumor cell phenotype [4]. In addition, we have very recently demonstrated improved anticancer effects using Braco-19 in combination with an oncolytic adenovirus by increased viral entry and replication in breast cancer cell lines [15].

The capacity of Braco-19 to induce cytotoxicity in ASCs during their differentiation into adipocytes was excluded in our experimental setting. However, the observation of morphological changes in cells undergoing differentiation and treated with Braco-19 suggested an interference of G4 motifs in the differentiation process. Additionally, the analysis of the cell cycle progression indicated a different behavior between undifferentiated and differentiated cells. The treatment with Braco-19 confirmed the absence of cell death and prevented G1 accumulation whose lengthening is known to accompany the differentiation [28], thus moving toward an undifferentiated-like profile in ADIPO. The investigation of the mechanisms involved in cell differentiation outlined that Braco-19 decreased lipid accumulation, cell dimension and complexity and reduced the expression of *PPARG, AP2* and *LEP* adipogenic markers, thus corroborating the hypothesis of involvement of G4 motifs during adipocyte differentiation.

G4 motif stabilization by Braco-19 has been associated with DNA damage-induced senescence in cancer cells [3]; however, in differentiating ASCs, the treatment with Braco-19 did not significantly affect β-gal activity and *LMNB1* mRNA expression, thus suggesting that the progress toward a physiological senescent phenotype of differentiating adipose cells is not strictly related to G4 motifs. Accordingly, *ACTA2* and *FSP1* fibrotic markers were not modified by Braco-19 treatment in ADIPO.

We also analyzed the impact of Braco-19 on inflammatory markers during adipocyte differentiation of ASCs. Among pro-inflammatory genes analyzed, we detected reduced *TNFA* mRNA levels following Braco-19 treatment, while *IL-6, IL-8* and *VEGF* expression was not significantly affected. However, although not statistically relevant, a decreasing trend of *VEGF* mRNA levels seemed to be observed.

A very recent study disclosed the analyses of putative quadruplex forming sequences (PQS) in inflammatory mediators [19]. In particular, the promoters of a number of cytokines and chemokines displayed high PQS frequencies comparable to those detected in proto-oncogenes. Among these genes, *TNFA* contains PQS with high propensity to form stable G4 structures, thus suggesting a role of these sequences in the function and regulation of this gene. Unfortunately, however, possibly due to small absolute amounts in ADIPO, secreted TNF-α protein levels were undetectable.

Similar as mRNA levels, IL-6 and IL-8 secretion was not modulated by Braco-19. Nevertheless, VEGF levels were significantly down-regulated. Indeed, it was demonstrated that adipocyte differentiation in bone marrow MSCs is controlled by intracellular VEGF by regulating *PPARG* expression [29]. Accordingly, in our cell model Braco-19 reduced the secretion of VEGF paralleled by a down-regulation of *PPARG* expression, further supporting a regulatory role for VEGF.

Notably, the formation of G4 motifs was revealed in the promoter of *VEGF* [20] and in *VEGFR2* [30]; the latter study also showed that the use of G4 binders inhibited *VEGF* expression and endothelial cell functions.

The analysis of G4 motif abundance showed up-regulation of G4 structures in ADIPO compared to ASCs. Interestingly, Braco-19 reduced the amount of G4 motifs in ADIPO, in contrast with the widely reported activity of that molecule as a stabilizer of the G4 structures.

Overall, our results show that destabilization of G4 motifs by Braco-19 during adipocyte differentiation of ASCs impairs cell differentiation and is accompanied by reduced *TNFA* mRNA levels and secretion of VEGF. A very recent study showed G4 motif abundance in hESC and reduction upon their differentiation into cranial neural crest cells and neural stem cells [9]. Additionally, it described that gain or loss of G4 structures at promoter regions during differentiation was paralleled, respectively, by increased or decreased gene expression in daughter differentiated cells [9]. Our study reports increased G4 motif content in ADIPO compared to undifferentiated ASCs. This effect might be ascribed to a potential acquisition of G4 structures in promoters of specific genes during differentiation, or by G4 motif stabilization by specific factors. Of note, insulin growth factor-1 (IGF-1), a significant modulator of cell growth and differentiation, is secreted by differentiating ASCs [12, 31] and it was reported to selectively bind G4 motifs [32]. Thus, IGF-1 production might account for G4 motif stabilization. It could be hypothesized that Braco-19 may impair IGF-1 interaction with G4. Accordingly, other molecules, as TMPyP4 and pyridostatin, have been described to reduce G4 interaction with IGF-1 [32].

Additionally, it was reported that the use of small molecules able to stabilize G4 structures in hESC delayed their differentiation, maintaining them in a more pluripotent-like status [9]. Differently, in our study, the destabilization of G4 structures by Braco-19 was accompanied by a reduced cell differentiation. Collectively, these data highlight a role of G4 motif modulation during cell differentiation.

Of note, a study employing surface-enhanced Raman scattering demonstrated the involvement of telomerase in the proliferation of human MSCs and in their differentiation to neural stem cells [33], thus supporting the hypothesis that telomerase is required for cell differentiation. In particular, telomerase activity showed higher expression until the third generation of human MSCs and then faded away [33]. Braco-19 was designed to stabilize G4 structures that may form within telomeric DNA, thereby inhibiting the access to telomerase [34–36]. Thus, we might speculate that in our experimental model, Braco-19 behaving as a G4 motif destabilizer might induce telomerase activity redirecting ADIPO toward undifferentiated ASCs. However, further studies are needed to confirm our hypothesis.

The investigation of the mechanisms of adipocyte differentiation in ASCs might provide better understanding of the pathogenesis of metabolic disorders, such as obesity and diabetes. High glucose conditions were reported to promote lipid accumulation into differentiating human MSCs up-regulating mRNA expression of adipogenic (*PPARG, FABP-4, CREBP alpha and beta*) and inflammatory genes (*IL-6* and *TNFA*) [37]. Indeed, previous studies reported that heterozygous *PPARG*-deficiency protected mice from the development of insulin resistance due to adipocyte hypertrophy under a high-fat diet [38]. In contrast, the subcutaneous adipose tissues of obese individuals and patients with type 2 diabetes display reduced PPARG activity [39], and PPARG activation in adipocytes was reported to be sufficient to reduce insulin resistance [40], hallmark of hypertrophic obesity. In this case, the production of new adipocytes might prevent the increase in large insulin-resistant adipocytes [41]. In this context, our data showing down-regulation of PPARG, AP2, LEP and TNFA mRNA levels by Braco-19 treatment of differentiating ASCs suggest that targeting G4 motif might be beneficial against obesity-associated excessive lipid accumulation and inflammation. Overall, understanding the role of MSC adipogenesis in obesity and related co-morbidities needs to be widely investigated. Interestingly, a recent study using micro-RNA-26a (miR-26a) knock-in and knock-out mouse models reported that RNA G4 motifs impair the maturation and function of miR-26a [42]. Mir-26a, known to induce pathways related to energy dissipation [43], is reduced during obesity, thus contributing to insulin resistance and metabolic diseases. In the same study, the use of DEAH-box helicase 36 (DHX36), binding and unwinding G4 structures, promoted miR-26a maturation. This study suggests that RNA G4s can regulate insulin sensitivity and lipid metabolism [43].

## Conclusions

Overall, our work highlights a new role of G4 motifs as genomic structural elements related to human ASC differentiation into mature adipocytes.

We might hypothesize that targeting G4 motifs may be beneficial for example in metabolic diseases. However, new investigations are required to clearly highlight the role of these structures in physio-pathological processes.

## Data Availability

Datasets are presented in the main manuscript.

## Abbreviations

G4: G-quadruplex
ASCs: Adipose-derived mesenchymal stem cells
*UCP1*: Uncoupling Protein-1
TMPyP4: 5,10,15,20-Tetra(*N*-methyl-4-pyridyl) porphyrin
RHAU: RNA helicase associated with AU-rich element
hESC: Human embryonic stem cells
MSCs: Mesenchymal stem cells
ADIPO: Adipocytes
SRB: Sulforhodamine B
PPIA: Peptidylprolyl isomerase A
qPCR: Quantitative real-time PCR
FSC: Forward scatter
SSC: Side scatter
FSC-H: FSC height
FSC-A: FCS area
MFI: Mean fluorescence intensity
SASP: Senescence-associated secretory phenotype
PQS: Putative quadruplex sequences
miR-26a: Micro-RNA-26a
DHX36: DEAH-box helicase 36
